# Win-Win for Wind and Wildlife: A Vision to Facilitate Sustainable Development

**DOI:** 10.1371/journal.pone.0017566

**Published:** 2011-04-13

**Authors:** Joseph M. Kiesecker, Jeffrey S. Evans, Joe Fargione, Kevin Doherty, Kerry R. Foresman, Thomas H. Kunz, Dave Naugle, Nathan P. Nibbelink, Neal D. Niemuth

**Affiliations:** 1 Global Conservation Lands Program, The Nature Conservancy, Fort Collins, Colorado, United States of America; 2 North America Conservation Region, The Nature Conservancy, Minneapolis, Minnesota, United States of America; 3 Audubon Society, Laramie, Wyoming, United States of America; 4 Division of Biological Sciences, University of Montana, Missoula, Montana, United States of America; 5 Department of Biology, Center for Ecology and Conservation Biology, Boston University, Boston, Massachusetts, United States of America; 6 College of Forestry and Conservation, The University of Montana, Missoula, Montana, United States of America; 7 Warnell School of Forestry and Natural Resources, University of Georgia, Athens, Georgia, United States of America; 8 United States Fish and Wildlife Service, Bismarck, North Dakota, United States of America; University of Kansas, United States of America

## Abstract

Wind energy offers the potential to reduce carbon emissions while increasing energy independence and bolstering economic development. However, wind energy has a larger land footprint per Gigawatt (GW) than most other forms of energy production, making appropriate siting and mitigation particularly important. Species that require large unfragmented habitats and those known to avoid vertical structures are particularly at risk from wind development. Developing energy on disturbed lands rather than placing new developments within large and intact habitats would reduce cumulative impacts to wildlife. The U.S. Department of Energy estimates that it will take 241 GW of terrestrial based wind development on approximately 5 million hectares to reach 20% electricity production for the U.S. by 2030. We estimate there are ∼7,700 GW of potential wind energy available across the U.S., with ∼3,500 GW on disturbed lands. In addition, a disturbance-focused development strategy would avert the development of ∼2.3 million hectares of undisturbed lands while generating the same amount of energy as development based solely on maximizing wind potential. Wind subsidies targeted at favoring low-impact developments and creating avoidance and mitigation requirements that raise the costs for projects impacting sensitive lands could improve public value for both wind energy and biodiversity conservation.

## Introduction

Within the United States, the world's largest cumulative producer of greenhouse gases, societal concerns have shaped energy policy supporting a dramatic increase in wind energy generation. The Department of Energy's (DOE) envisions the U.S. producing 20% of its electricity from wind by 2030, as outlined in their report “20% Wind Energy by 2030,” hereafter “20% vision” [1]. However, wind energy has, per unit energy, a larger terrestrial footprint than most other forms of energy production [2], [3] and has known and predicted adverse impacts on wildlife [4]–[7]. Meeting the DOE 20% vision (∼241 Gigawatts of on-shore wind with an additional 64 Gigawatts of off-shore wind) would result in 5 million hectares of impacted land, an area roughly the size of Florida, with an additional 18,000 kilometers of new transmission lines [1]. While wind generation remains small as a percentage of electrical output in the United States, it is one of the fastest-growing renewable energy sectors, with more than 35.6 GW of installed capacity as of March 2010 [3]. This growth is manifested in arrays of turbines that cover large areas, as each turbine generates relatively little power compared to conventional sources. Wind “farms” have a broad footprint and thus are highly susceptible to land use conflicts common among other forms of energy development. While environmental concerns over wind development have focused primarily on direct strike mortality of birds and bats [4]–[7] it is the increase in fragmentation and habitat loss associated with development that creates an important conservation challenge [7]. In the U.S. the Federal Endangered Species Act currently protects over 1300 species and another ∼250 species are under consideration for protection. The majority of these species list habitat loss and fragmentation as the primary cause for federal protected status (http://www.fws.gov/endangered/). Siting of wind development that avoids habitats important for biodiversity reduces the potential for significant habitat loss and fragmentation and corresponding listing of additional species.

**Figure 1:**
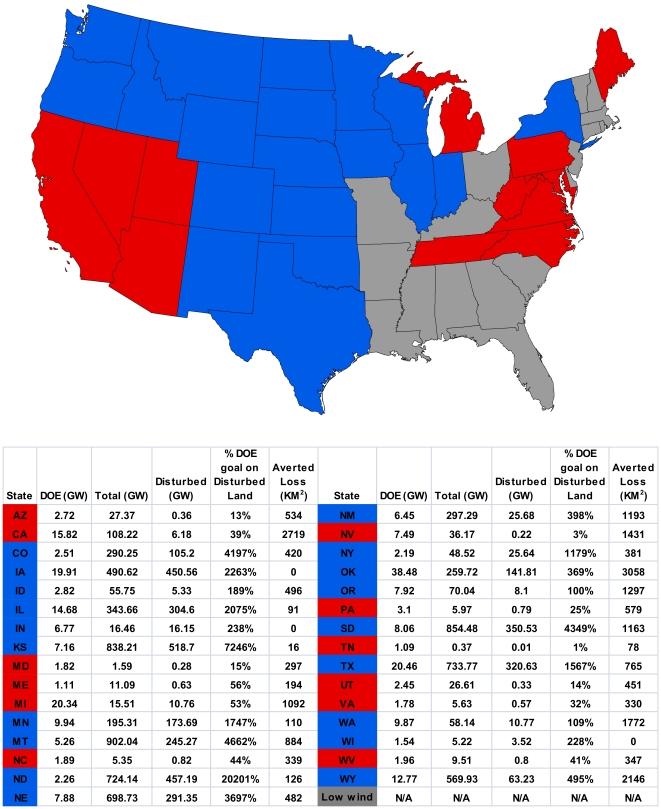
Map of continental U.S. with states where DOE targets can (blue) and cannot (red) be met on disturbed lands. We focused on the 31 states that comprise the majority of the DOE vision, excluding states (grey) with less than 1 GW of projected development [1]. Inset table with 31 focal states, their DOE projections (in GW), Total available wind energy (in GW), wind energy available on disturbed lands (in GW), percent of DOE vision that can be met on disturbed land and amount of undisturbed lands that a disturbance focused development scenario would avert (in square kilometers).

In this study we examine patterns of wind energy potential in terrestrial landscapes that are already disturbed by human activities (e.g., agriculture, oil and gas development). Although other studies [8] have estimated the total amount of potential wind-energy production available in the U.S. and globally, this is the first to examine if renewable energy goals can be met on disturbed lands that could reduce conflict with wildlife. Our goal is to estimate the potential electricity generation capacity of lands of low value for biodiversity conservation rather than estimate impacts associated with wind farms and associated transmission. Our scenarios (Figure 1) are based on the DOE forecast of wind energy production for each state to meet the 20% vision [1]. The DOE projections outline a spatial and temporal roadmap for meeting wind energy goals, with specific GW projections for each of the lower 48 states. Here we focus on the 31 states that comprise the majority of the DOE 20% vision, excluding states that have ≤1 GW of projected development: AL, AR, CT, DE, FL, GA, KY, LA, MA, MO, MS, NH, NJ, OH, RI, SC, VT [1]. We calculated the area needed to meet DOE wind energy scenarios within each state, providing a broad overview of the potential for wind energy generation on disturbed lands, but did not attempt to predict where within each state wind energy development will take place. The land area needed to meet the 20% vision depends on the wind potential of any given area, as characterized by its wind power class [9]. Foregoing development of undisturbed land with high wind classes in favor of disturbed lands (with potentially lower wind classes) may require more land to generate the same amount of energy. Therefore, we examined if meeting DOE goals solely on disturbed lands would require an increase in land area over that needed when the highest wind classes are exploited regardless of disturbance. Finally, we discuss the likelihood that targeting development to already disturbed lands will reduce impacts to biodiversity, and potential limitations to this conclusion.

## Results

Croplands cover 1,954,821,517 ha, planted hay/pasture 521,779,323 ha, impervious surfaces 380,885,661 ha, oil and gas fields 365,236,244 ha, surface mines 1,212,619 ha and urban-developed lands 480,230,891 ha. Total disturbed lands were 3,218,665,150 ha, with some disturbances overlapping. After removing urban areas, permanently protected lands, and areas with wind power classes less than three, there were 1,450,443,444 ha considered suitable for wind.

**Figure 2:**
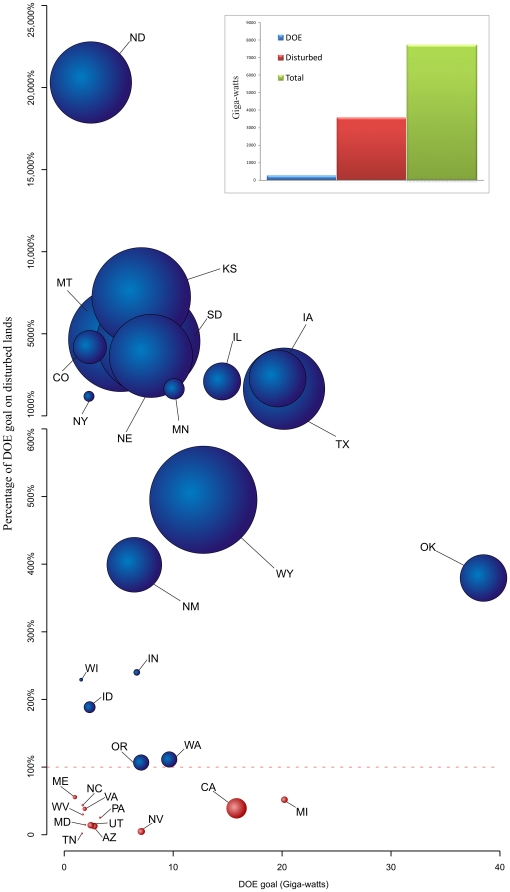
Available wind-generated Giga-watts (GW) in each state as a function of the DOE goal and percentage of the DOE goal that can be met on disturbed land. Bubbles indicate where DOE goals can (blue) and cannot (red) be met on disturbed lands. Bubble area indicates total GW of wind potential available in the state (Range 0.37 GW in TN to 902 GW in MT). Inset graph shows potential GW wind production for the entire U.S. and potential on disturbed lands relative to the DOE 20% projection.

**Figure 3:**
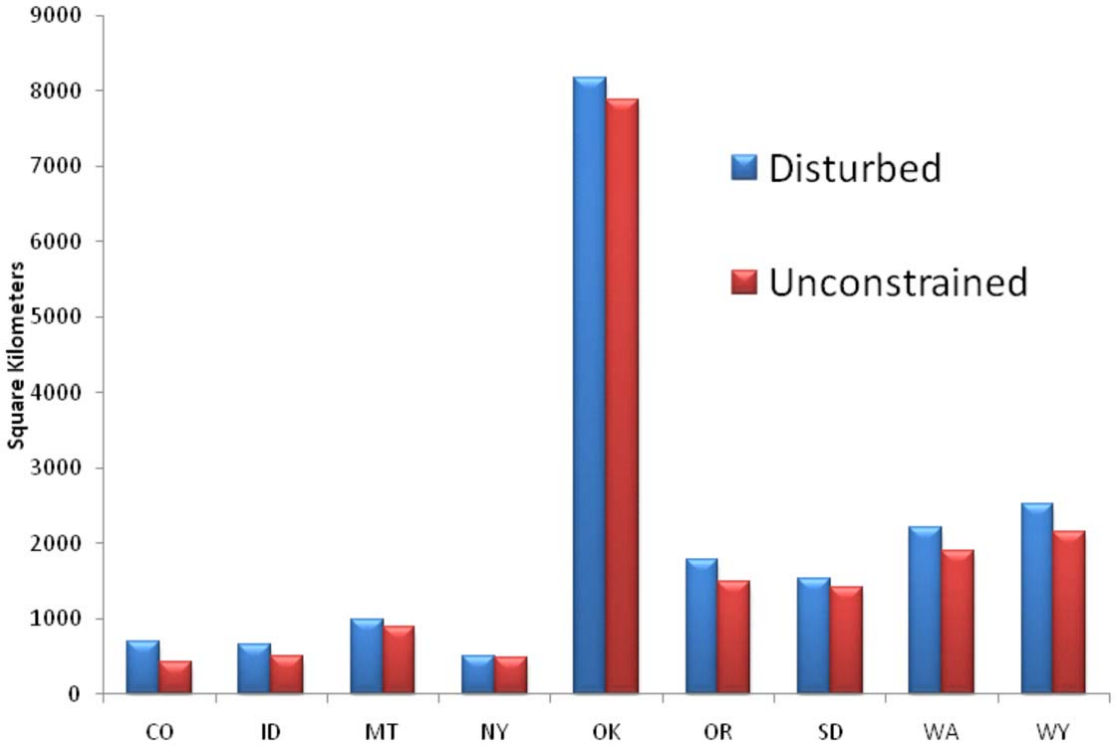
Minimum number of square kilometers needed to meet DOE projections for disturbance restricted (blue) or unconstrained (red) scenarios. For simplicity we have only included states where disturbance focused development would result in an increased area needed to meet the DOE projections. For all other states there is either not an increase in land needed or the state is unable to meet DOE projections on disturbed lands.

Our analysis indicates that a network of land-based turbines, accounting for areas inappropriate for their placement, has the potential to generate 7,705 GW in the lower 48 United States, with potential for 3,554 GW in areas already disturbed by human activities (Figure 2). Given a DOE projection of 241 terrestrial GW, there is ample opportunity to meet this goal in areas likely to have relatively low wildlife value. Despite the extensive wind resources across the U.S., nine states (CA, AZ, NV, UT, WV, PA, VA, NC, & TN) are unable to meet DOE projections within areas already disturbed (Figures 1 & 2). There are also three states (MD, MI & TN) that are unable to meet DOE terrestrial projections even if wind development is not confined to disturbed lands. Given the distribution of wind power classes, an additional nine states (CO, ID, MT, NY, OK, OR, SD, WA & WY) would require an increased land base to generate the same amount of GW if development is focused solely on disturbed lands (Figure 3). Notwithstanding these tradeoffs, a disturbance-focused development strategy would avert the conversion of ∼2.3 million hectares of undisturbed lands relative to the unconstrained scenario in which development is based solely on maximizing wind potential.

## Discussion

Shifting energy production from fossil fuels to renewable energy that collects more diffuse energy from a broader spatial area will involve tradeoffs. Wind energy production will result in reduced CO_2_ emissions and reduced water demand for electricity generation [1], but it will result in broader terrestrial [2], [7] and aerial impacts [4]–[6]. The increase in wind production forecasted by DOE may be compatible with wildlife if properly sited, but will still pose a challenge for conservation, both because of the threat of bird and bat mortality [4]–[6] and because of the large area impacted, which may cause habitat loss, fragmentation, and avoidance [7], [10], [11]. There are multiple ways to balance the tradeoffs between emissions reduction and increased fragmentation resulting from wind energy development. First, energy conservation can help reduce the new energy needed by the U.S., reducing the area impacted by new energy development [2]. Many impacts can be mitigated or eliminated with appropriate siting and planning for energy development [12]. Planning for the siting and mitigation of industrial scale wind development will require that we examine tradeoffs at an appropriate landscape scale. We contend that identification of large areas of disturbed land represent the first step in a series of hierarchical filters that can guide wind development to reduce impacts to wildlife species. Harnessing the power of systematic conservation planning [12] will allow stakeholders to examine cumulative impacts associated with wind and other development as well as balance other land use needs and issues (e.g. view sheds) that will be important in addition to wildlife.

The disturbed areas used in this analysis represent low-quality habitats incapable of supporting populations of imperiled species and are altered to the point of no longer supporting natural community assemblages [13], [14]. Disturbance is also consistently associated with reduced biological integrity and increased probability of extirpation for many species [15], such that areas of high disturbance generally have low value for biodiversity [16]. Patterns of disturbance have historically played a significant role in the design and development of conservation priorities [13], [16]. From a conservation perspective, the types of species that accumulate in disturbed landscapes do not compensate for the loss of biodiversity resulting from fragmentation of once large and intact landscapes [17], [18].

Conversely, areas of low disturbance are disproportionately valuable for biodiversity. Species of conservation concern that require large intact shrubland or grassland habitats, such as sage grouse and greater and lesser prairie chickens, are sensitive to human activity and may be evolutionarily adapted to avoid large vertical structures such as wind turbines, and are therefore thought to be particularly vulnerable to wind energy development. These grouse exhibit 90% reduction in nesting up to 1.25 miles away from vertical structures such as wind turbines [10]. For these and other species that require large unfragmented habitat, improperly sited wind turbines may be incompatible with maintaining viable wild populations.

The approach we outline here is not intended to prescribe exactly where turbines should be located, but instead to demonstrate that there are many options for wind development. Site- specific characteristics or landowner preferences may limit the ability to develop any particular piece of disturbed land. However, given the large area of disturbed lands that have suitable wind resource, most of the projected wind development in the U.S. could be targeted onto existing disturbed lands. New wind development would likely have minimal potential to impact terrestrial wildlife if sited in disturbed areas. In addition to reduced wildlife impacts, a disturbance-based development strategy is largely compatible with current land uses. For example, given turbine spacing needs, wind farms typically utilize only 2–4% of an area, making it compatible with agricultural production [19]. Moreover, compensation associated with development increases profitability of lands that balance agriculture and wind development [1]. While land in corn production yields profits of less than $1,000 per ha [20], farmers may receive $4,000–$6,000 per year per turbine [21]. A turbine and associated infrastructure have a per-turbine footprint of less than one ha, thus farmers receive more than adequate compensation to encourage them to convert some of their (already disturbed) cropland to wind energy development. The other types of disturbance used in our analyses are also physically compatible with wind development, within or adjacent to these lands. Although wind development on oil and gas fields is currently often limited by land rights and competing interests, these two forms of development are physically compatible and co-location could be facilitated and incentivized with targeted policies and subsidies. Agriculture and oil and gas make up the vast majority of the disturbed lands identified in our analysis, such that removal of other disturbed lands would not qualitatively change our results. However, we believe that ridges surrounding abandoned surface mines and areas adjacent to existing roads also constitute disturbed areas where wind energy development should be considered.

Placing turbines on disturbed lands may also benefit the expansion of transmission lines and associated infrastructure that will be critical to facilitate wind development. Because disturbed lands are already in areas of high road and transmission line density, they may ease the development of new or expanded transmission capacity. As transmission capacity is expanded, consideration should be given to its design to ensure its placement considers wildlife conservation and can encourage development of wind on disturbed lands. Given the nationwide surplus in wind energy, it is conceivable that states that cannot meet goals on disturbed lands could import electricity from states where there is a surplus of disturbance based wind energy. A number of states (MT, SD, KS, TX, ND, NE, WY, IA & IL) have a significant surplus of wind potential on disturbed lands where additional development would not likely cause significant loss of wildlife (Figure 2). Moving development to states where there is a surplus of wind potential on disturbed lands may alleviate some of the conflict over impacts to wildlife, if feasible given transmission and political constraints.

Targeting state and federal subsidies to favor low-impact developments and creating avoidance and mitigation requirements that raise the costs for projects impacting undisturbed lands could maximize public value for wind energy and wildlife conservation. Steering development to already disturbed landscapes may increase the spatial extent of wind energy (Figure 3) but will also decrease resulting impacts to wildlife by limiting habitat fragmentation (Figure 1). For example, in the nine states where wind development sufficient to meet the DOE target on disturbed lands requires more turbines, only increases the land area required to meet the 20% vision in these states by 11%, an increase of less than 2,000 km^2^. We recognize that in these nine states a disturbance-focused development strategy may require increased investment to produce the same amount of electricity. However, as wind development increases, conflicts over impacts to wildlife are likely to become increasingly important. Thus, a proactive approach that seeks to avoid impacts to wildlife will reduce overall costs and facilitate wind development.

Several caveats limit our ability to conclude that a given disturbed area has low wildlife values. First, we are measuring terrestrial disturbance, which may not be correlated with use of the aerosphere by birds, bats, and insects [4]–[6]. In particular, birds require migratory stopover sites, and these may occur along rivers, wetlands, or playa lakes that are embedded within heavily disturbed agricultural landscapes. Second, even terrestrial species may require migratory corridors through disturbed areas to access undisturbed habitat. Although currently quantitative nationwide data on airspaces with high bird/bat use do not exist, available regional and local information on migratory corridors, stopover sites, and aerospace use will be important to incorporate into local siting decisions. Additional research on land-cover and landscape features associated with bird and, particularly, bat mortality is needed to confidently identify areas where wind development would cause low mortality. In spite of this limitation, several factors suggest that a disturbance based approach to wind siting will reduce overall impacts to wildlife. First, strategies other than siting may be the most appropriate for addressing bird and bat strike mortality. For example, mitigation measures, such as feathering blades (which stops their rotation) or reducing operations during lower winds speeds when bat mortality is known to be high (fall migration nights when wind speeds are less than 5.5 m/s) could reduce bat mortality independent of where wind energy is sited [22], [23]; micrositing of turbines can reduce bird mortality [24]. These strategies can be applied on both disturbed and undisturbed lands. Second, there is no reason to expect that siting wind turbines on disturbed lands would increase direct mortality to birds and bats. Even in cases where targeting disturbed lands requires the use of lower wind power classes and therefore more turbines to produce the equivalent amount of energy, these turbines would have reduced movement (i.e. would spend a smaller fraction of the time moving). It is likely that mortality at turbines that are not moving will be negligible [25]. Finally, even with 241 GW of on-shore wind energy, wind energy would kill less than 1 million birds per year. This is a very small proportion of the direct human-caused mortality to birds, which has been estimated at 300–2,300 million birds per year due to (in descending order of importance) cats, windows in buildings, poison, transmission lines and communication towers, cars, and oil and waste water pits [26]. At worst, wind energy would be responsible for a fraction of a percent of all human-caused bird mortality, although bat mortality has the potential to be have a much more significant population-level impact. Because species of conservation concern are preferentially found in native habitat versus cropland and other disturbed areas [14], [27], we expect that targeting wind energy development in disturbed areas would be more likely to impact birds that are not of conservation concern. In total, we believe that the identification of large areas of disturbed lands that are suitable for wind energy development and the targeting of wind energy and transmission line construction in these areas offer the potential to dramatically reduce the wildlife impacts associated with increased wind energy generation.

Our analysis may under-estimate the amount of wind resources available on disturbed lands. To estimate wind production potential we utilized 50-meter above ground wind data that is publically available and was used by the DOE to create the 20% vision [1], [9]. However, current turbine design places wind turbine hub heights at 80 meters where wind speeds are higher, allowing the economic development of wind on disturbed lands not identified as suitable in our analysis. Further, our analysis may not identify all areas with disturbed lands. Although we have a high degree of confidence in our ability to predict areas impacted by disturbance, we recognize that areas characterized as undisturbed in our analysis do not represent “pristine wilderness”. Although undisturbed areas are free of overt disturbance, they may be impacted by other factors (i.e., invasive weeds) or other land-use practices that reduce the wildlife value (i.e., over-grazing), neither of which are included in our definition of disturbed areas. This suggests that there may be moderately disturbed areas that are suitable for wind development but are not captured in our analysis, although disturbance caused by poor land management can often be addressed through management and/or policy changes within otherwise intact and functioning ecosystems. In total, our estimates of the potential for developing wind on disturbed lands is likely conservative, such that the potential for avoiding impacts to biodiversity is even greater than indicated by our analysis.

Avoiding impacts to undisturbed areas will be critical to maintain wildlife in the face of climate change and future development [28]. Given the uncertainties inherent to planning for long-term conservation goals with a shifting climate and the potential for strong interactions between climate change and other stressors, many have recognized the need to develop adaptation strategies to proactively mitigate the needs of wildlife conservation. Guiding development toward areas with existing footprints may represent the best opportunity to mitigate impacts associated with climate change [29]. Maintaining large and intact natural habitats and maintaining or improving the permeability of land for the movement of both individuals and ecological processes may provide the best opportunity for species and ecological systems to adapt to changing climate [28], [29]. The push to develop renewable energy is motivated in part due to the negative impacts that climate change would have on biodiversity. However, the potential benefits to biodiversity from climate change mitigation will be realized only if renewable energy development can avoid and mitigate impacts to remaining habitat [30]. Our analysis provides a first step toward a national blueprint to facilitate sustainable wind development in a manner that maintains areas important for wildlife.

## Materials and Methods

To develop a disturbance data layer that is relevant and comparable across the conterminous United States we utilized data that were consistently derived across large geographical/regional scales [31]. We used the National Land Cover Dataset (NLCD), classified into disturbed lands using the following classes: Cultivated Crops, Developed-High Intensity, Developed-Low Intensity, Developed-Medium Intensity, Developed-Open Space and Hay/Pasture (http://landcover.usgs.gov/natllandcover.php). We recognize that rangelands often serve as important wildlife habitat despite their intensive use by domesticated livestock. For this reason we have excluded these lands in our index. We used a Landsat™ derived impervious surface classification [32] to identify areas with reduced percolation, such as pavement. The USGS topographic change dataset (http://topochange.cr.usgs.gov/) was used to identify mines and other major human-based changes in topography. Oil and gas fields were integrated into the analysis using IHS energy© data [33]. While we have confidence in the ability of individual data layers to accurately predict disturbance patterns, misclassification error for individual data layers can be found in their respective data sources. We created a binary disturbance dataset by defining any 30-meter pixel classified as disturbed across the four independent datasets (landcover, mined, impervious, and oil & gas) as disturbed, otherwise undisturbed. We calculated the square kilometers (km^2^) of each wind power class within each state [9]. Following the DOE 20% vision [1], we estimated the amount of GW per unit area across the U.S. and in each state by assuming that average nameplate capacity (44.5%) is installed at 11.24 MW/km^2^ and adjusting turbine nameplate capacity with capacity factors specific to each wind power class (WPC 7 = 53%; WPC 6 = 49%; WPC 5 = 46%; WPC 4 = 43%; WPC 3 = 38%). These area requirements assume efficiency increases as predicted for 2030 by the DOE [1].

We estimated the amount of land in each state needed to meet DOE projections by selecting the largest contiguous blocks of disturbed lands in the highest wind power class in that state and repeating that process in successively smaller disturbed patches and lower wind power classes until the DOE projection was reached. The smallest patch sizes selected were all within the size range of existing or proposed wind developments. To generate an “unconstrained” development scenario, we repeated this process without restricting the selected areas to disturbed lands. We compared the amount of land needed to meet the DOE projection under the disturbance restricted and unconstrained scenarios. Once the land area needed to meet the DOE projection was determined, we measured the amount of undisturbed land that would need to be developed to meet goals in the unconstrained scenario. Throughout our analysis, we excluded certain areas as being protected or restricted from development, modeling our decision rules on those used in the DOE's report [1]. We excluded areas having a protected status precluding wind development using the Gap Analysis Program code 1 or 2 (i.e., permanent protection excluding development), based on the Protected Area Database of the United States, version 1.1 [34]. To avoid counting areas where land is not protected but large-scale wind will likely not be developed, we excluded urban-core areas [35], and wetlands and water bodies identified in the NLCD data. All spatial analyses were performed in ESRI's ArcGIS 9.3 (http://www.esri.com/) and all statistical analyses were performed in R [36].
